# Falling Detection of Toddlers Based on Improved YOLOv8 Models

**DOI:** 10.3390/s24196451

**Published:** 2024-10-06

**Authors:** Ziqian Yang, Baiyu Tsui, Jiachuan Ning, Zhihui Wu

**Affiliations:** 1 College of Furnishings and Industrial Design, Nanjing Forestry University, Nanjing 210037, China; 2 Jiangsu Co-Innovation Center of Efficient Processing and Utilization of Forest Resources, Nanjing 210037, China; 3 Qingdao Grace Chain Software Ltd., Qingdao 266071, China

**Keywords:** fall detection, toddlers, YOLOv8, pose estimation

## Abstract

If toddlers are not promptly checked and rescued after falling from relatively high locations at homes, they are at risk of severe health complications. We present a toddler target extraction method and real-time falling alarm. The procedure is executed in two stages: In stage I, a GELAN-integrated YOLOv8 model is used to extract the body features. Based on this, a head capture technique is developed to obtain the head features. In stage II, the “safe zone” is calculated through Generalized Hough Transform (GHT). The spatial location is compared to the preceding stage’s two centers of mass points, K for the toddler’s body and H for the head. Position status detection is performed on the extracted data. We gathered 230 RGB-captured daily videos of toddlers aged 13 to 30 months playing and experiencing upside-down falls. We split 500 video clips (×30 FPS) from 200 videos into 8:2 training and validation sets. A test set of 100 clips (×30 FPS) was cut from another 30 videos. The experimental results suggested that the framework has higher precision and recall in detection, as well as improved mean average precision and F1 scores compared to YOLOv3, v5, v6, and v8. It meets the standard FPS requirement for surveillance cameras and has an accuracy of 96.33 percent.

## 1. Introduction

Falling is a natural aspect of a child’s development as they learn motor and cognitive skills. It is especially prevalent when learning to walk, climb, jump, and explore their surroundings [1]. As children grow older, they are more likely to engage in challenging activities, sometimes called “risk-taking behaviors.” The Injury Review of Chinese Children and Adolescents [2] reveals that the mortality rate from falls among children aged 1–4 is disturbingly high, with falls being the leading cause of death in this population. The danger of falling is increased by up to eight times due to immature skeletal systems and a lack of self-protection habits in this age range. The Report on the Epidemiological Review of Injuries to Children and Youth in China [3] states that 46.34% of all cases of injuries occur at home. Falls account for the majority of injuries at home (40.18%). The most common falls among children, mainly those ages 1–3, are from stairs, baby walkers, furniture, or play equipment.

This paper focus on vision-based techniques for fall detection because of their benefits over wearable sensors. Vision-based techniques are less intrusive and eliminate the motion disturbance of toddlers wearing specific test clothes. With the proliferation of cameras in modern cities, we can expand the applicability of fall detection models from smart homes to public spaces and beyond.

The visual equipment mainly consists of several types of cameras, such as depth, thermal, and monocular. Mecocci et al. [4] employed a depth camera to detect falls in hospital rooms, with a sensitivity of 82.1% and a specificity of 97.8%. Booranrom et al. [5] utilized a Kinect camera to analyze ten volunteers’ falls and achieved 97% accuracy. Song et al. [6] suggested a thermal camera-based monitoring system, tested it on five volunteers, and evaluated the thermal images using SVN with a 99.7% accuracy. Kittipanya-Ngam et al. [7] monitored falls by recognizing the bed’s position relative to the person’s head. However, the method only recognizes the edges of the bed. It identifies the head features by skin color, which may cause serious errors. Banerjee et al. [8] estimated the location of the bed-ridden person by determining the person’s area, which reduces misjudgment of fall detection. Still, the study could not provide early warning of the falling behavior. Ni et al. [9] similarly proposed a monitoring method that utilizes visual information from RGB-D for fall warnings. Brulin et al. [10] perform fall detection using a gesture recognition algorithm based on fuzzy logic analysis. Still, due to the specificity of the algorithm and the camera, it cannot work during the night, which results in it being unable to address real-life applications. Two-dimensional cameras are a more cost-effective alternative to three-dimensional range sensors.

Takeda et al. [11] used NeNet to determine the relationship between humans and beds. Still, this method required complex parameter settings during picture segmentation processing, resulting in low computing efficiency and preventing real-time monitoring. Shen et al. [12] recognized the human head using template matching before extracting the entire body using region growth. However, this technology necessitates a more static stance for the human body and cannot accurately detect changing movements. Zhao et al. [13] detected the human head first and used its position to collect information about the upper half of the human body. However, if the subject’s body is obscured, information about the upper body cannot be accurately extracted, and the system’s operation time is considerable, making real-time monitoring impossible. Other studies [14,15,16,17] primarily identify the human body following a fall. They detect the person lying on the floor and do not consider dynamic information, making it difficult to discern between typical lying and accidental falls.

Researchers modified the feature extraction module’s design to enhance detection accuracy. Zhao et al. [18] adds the SDI (Shuffle Dimensionally Integrated) attention module to the Efficient Layer Aggregation Networks(ELAN) in the Backbone and Spatial Pyramid Pooling, Cross Stage Partial Channel (SPPCSPC) in the head, enabling the YOLOv7 model to focus on key areas or typical features. Lyu et al. [19] improved the SPP network by incorporating an extra 1 × 1 max-pooling layer, resulting in notable improvements in detection performance. Chen et al. [20] improved the YOLOv5 model’s extraction ability by incorporating spatial attention and average pooling layers into the Efficient Channel Attention(ECA) and SPP networks, respectively. Pereira GA [21] analyzed YOLOv8 variations and found that YOLOv8m achieved a good mix of computational efficiency and detection performance. Sun et al. [22] used Single Shot Detector (SSD) MobileNet to remove the non-human key points detected by OpenPose, and the SVDD classification algorithm classifies the values. In summary, these studies have shown high accuracy, sensitivity, and specificity, but the datasets used are laboratory environments and mostly simulated activities of daily living (ADLs) and falls, which have significant limitations.

Creating datasets from publicly available data or real-world surveillance footage makes a lot of sense for training detection models. The approach should adapt to a wide range of surveillance images, including those with differing small distractions and occlusions in the video and varying ambient settings and lighting conditions. He et al. [23] use an attention-integrated YOLOv5-alike model to extract infant portraits. Then, a single-shot multi-box detector filters out spurious targets and accurately recognizes the position. The dataset was derived from actual surveillance captured in baby pools. Furthermore, additional actual surveillance was used as the test set. Marcos et al. [24] present a transformer-based fall detection method. They use transformers to extract characteristics from raw RGB frames, eliminating the need for further skeleton and position computations. They evaluate the fall detection model on the UP-Fall dataset [25] and on the UR fall dataset [26]. Qin et al. [27] replaced YOLOv8′s C2f module in the backbone network with the C2Dv3 module to capture more intricate features. The DyHead block was utilized in the Neck section to unify multiple attentions, increasing the detection accuracy of targets at different scales. Maudsley-Barton [28] employs an LSTM autoencoder to calculate a distance measure on the KINECAL dataset. Using this measure, he develops a new scoring system that places individuals with varying fall risks on a continuous scale.

In everyday scenarios, toddlers are prone to sudden and swift falls from furniture. Surveillance cameras integrated with the YOLO recognition algorithm offer an effective solution to these challenges. They are capable of capturing data on multiple individuals concurrently, eliminating the need for additional sensors. These cameras boast swift processing capabilities, facilitating real-time data analysis. Furthermore, they are economically viable and enhance the overall user experience.

Our study focused on toddlers, characterized by their disproportionately large heads and tendency to crawl and roll on furniture like beds and sofas. To accurately determine falls, we consider the combined center of mass for both the body and head in relation to the furniture edges.

**Figure 1:**
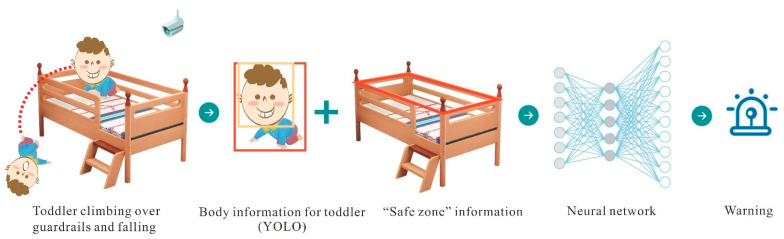
General structure of child falling detection system.

We propose a toddler target information extraction approach based on improved YOLOv8.This method can accurately recognize the target subject, even in cases where the person is overlapping with furniture. Simultaneously, given that the positions of furniture and the ground are typically unchanging, We use GHT to identify forms and extract the shape of the “safe zone” surface using a predefined template. We then calculate the positional relationship between the human body target, head target, and the “safe zone” to generate a real-time alarm for falling behavior, as depicted in Figure 1.

Due to the specificity of the toddler population, we first built our dataset by collecting 230 daily surveillance videos of toddler falls captured by RGB cameras. These videos primarily recorded the daily activities of 13- to 30-month-old toddlers playing near the edge of a piece of furniture or a guardrail before falling. The length of these videos varied from 10 min to 15 s. One hundred and eight parents agreed to use video surveillance videos for research purposes.

The main contributions are as follows:

1. This study use GHT to identify forms and extract the shape of the “safe zone” surface using a predefined template.
2. A new dataset of 500 video clips (×30 FPS) is created using 200 real-time daily videos collected from 100 parents. Another test set of 100 falling video clips (×30 FPS) is created using 30 real-time daily videos collected from 8 parents. The datasets collected from daily surveillance video feature varying lighting conditions, camera settings, furniture placement, and furnishing arrangement, increasing the diversity of the generated dataset and enhancing the framework’s generalization capacity.
3. Given that the standard YOLOv8 method is insufficient for collecting body and head information at higher precision, we have improved the original YOLOv8 program. We recommend replacing the C2f module in YOLO with the GELAN module. One advantage of GELAN is that it uses the convolutional network layer and can contain any computational module. It increases the overall flexibility of the network topology and allows it to fulfill the needs of our information extraction techniques successfully.
4. A real-time system is proposed to detect instances of toddlers falling in their home surroundings. This technology efficiently and precisely captures data of toddler targets in challenging environments, converting the human body into a rectangular shape, simplifying the data without sacrificing accuracy, and increasing the usefulness of real-time artificial intelligence for fall detection. It is an early warning preparation for a later head injury assessment. After thorough enhancement and streamlining, it may be deployed on the hardware infrastructure of smart home devices.

The remaining sections of this work are structured as follows. In Section 2, the detection approach’s details of toddlers falling are designed. Section 3 presents the conducted experiments and discusses their outcomes. The framework’s benefits and constraints are examined in Section 4. Conclusions are derived in Section 5.

## 2. The Proposed Methods

Toddlers are frequently put in flat areas of the home with guardrails and on beds and sofas. The primary challenge in monitoring toddlers’ falls from furniture is obtaining information about their bodies efficiently. Our studies have determined that OpenPose is unsuitable with our framework, even though we initially considered it for a comparative study together with YOLO. The main reasons are as follows:

1. Posture changes: Toddlers show a variety of postural modifications. Their limbs are often more flexible than adults, who often maintain an upright posture and move regularly. This variation can make it difficult to identify posture via skeletal analysis.
2. Differences in physical characteristics: Toddlers have shorter limbs, larger heads, and notably distinct proportions compared to adults. OpenPose may not capture these traits well, leading to imprecise detection outcomes.
3. Differences in the coherence of movement: Toddlers have less predictable and coherent motions and tend to move quicker and erratically, especially when lying down or crawling, making it challenging for the OpenPose model to track them effectively.
4. Problems with loose clothing covering: Toddlers typically wear clothing that fits loosely, which can conceal joints and make it more challenging to identify them.
5. The problem of real-time availability: OpenPose primarily captures human skeletal point data, and excess recognition data may cause interference. In real applications, the approach requires significant computational resources and processes at a slower speed.

Consequently, YOLO was ultimately selected to extract the body information of toddlers.

We carefully examined the toddler’s fall video without interruption to ensure that our analysis was accurate. YOLO-based body information extraction models received keyframes. This model accurately identified the child’s head and torso from the input image.

Following that, manual and automatic parsing techniques were used to thoroughly evaluate the furniture and floor, determining the optimal ‘safe zone’. Given the architecture of our technique, this procedure may be carried out in real-time on a conventional computer.

Finally, by examining the relative placements of these two zones and using a state delineation method, we may precisely define the child’s current state.

**Figure 2:**
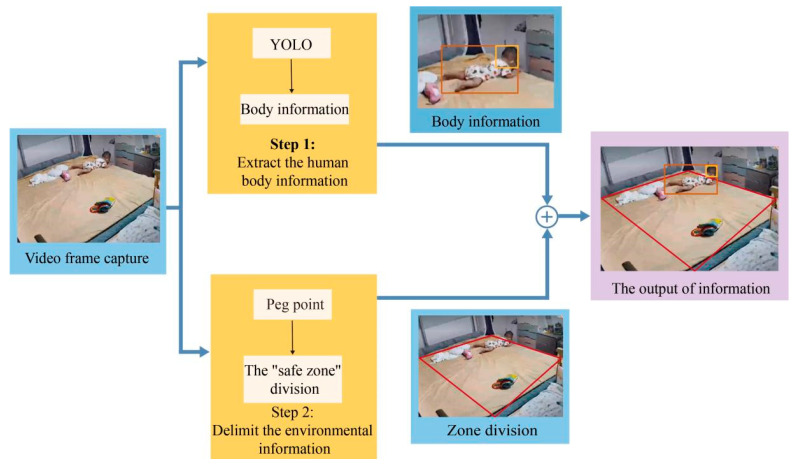
General structure of toddler falling detection system.

Our classification approach identifies whether a toddler is safe or at risk of falling. Using the previously mentioned basic methodology, we created an accurate target identification algorithm and a unique classifier for detecting potential hazards. Figure 2 depicts the workflow for our framework.

### 2.1. Extracting Body Information of Toddler

To effectively solve the issue, we propose a technique for extracting body information based on the YOLO algorithm. Subsequently, a Generalized Efficient Layer Aggregation Network (GELAN) is developed, utilizing an ELAN, to substitute the C2f module in the backbone network. This replacement is intended to improve the network’s ability to extract features and give a more lightweight model.

GELAN combines the Cross Stage Partial Network (CSPNet) and ELAN procedures, integrating RepConv to achieve more convincing results. The reasoning speed is unaffected when the network environment is minimally influenced due to the lightweight processing and tiny amount of data.

**Figure 3:**
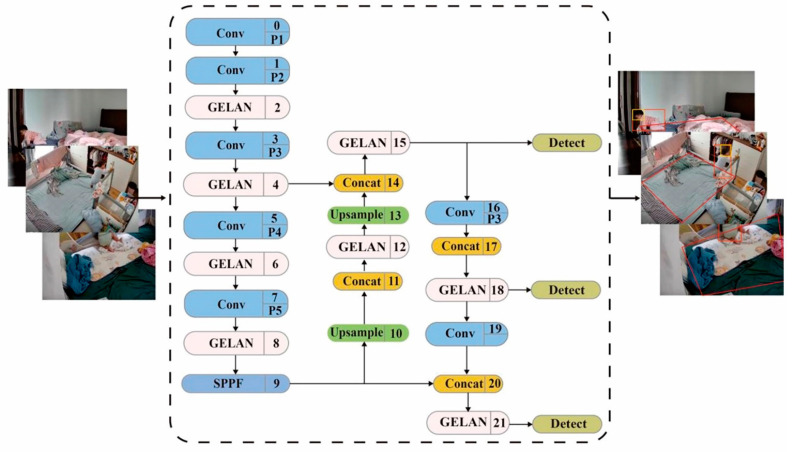
Overall structure of toddler’s body information extraction.

The human head is more recognized and has consistent visual traits than other body parts. As a result, our approach prioritizes detecting body information before retrieving a toddler’s head details. First, the child’s physical parts are properly identified. The head data are then retrieved and verified using positioning body information and a GELAN module to focus on the child’s head, an improved alternative to the C2f module in YOLO. Potential body areas are identified based on the spatiotemporal consistency seen between consecutive frames to maximize efficiency. Figure 3 displays the major technical route for retrieving information from child bodies.

**Figure 4:**
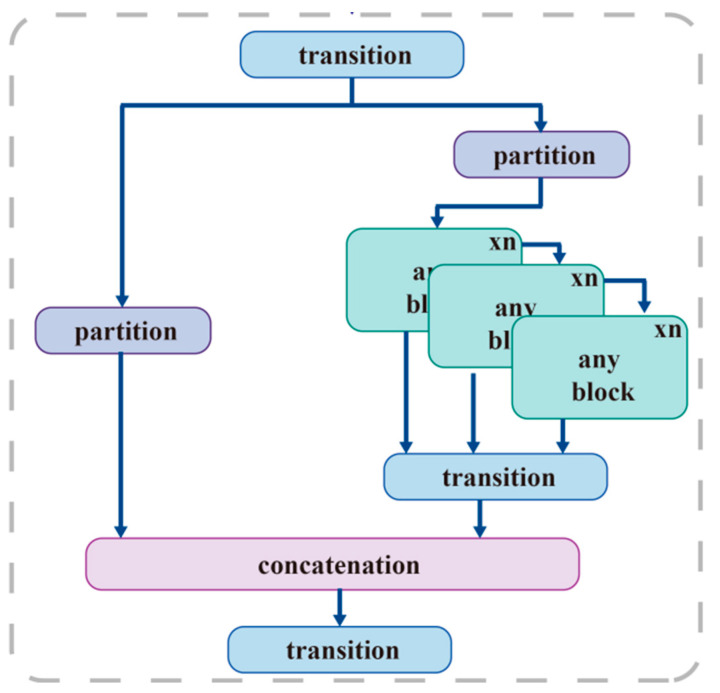
The GELAN module architecture.

The GELAN module, integrated into our framework, merges two neural networks: CSPNet and ELAN. Both are designed via gradient pathways, allowing us to combine their strengths into a GELAN. This network excels at lightweight construction, fast inference, and high accuracy. This makes it ideal for devices with little resources. Our framework extends the functionality of the ELAN architecture. The internal framework structure is shown in Figure 4.

GELAN integrates arbitrary computing blocks and a convolutional network layer. This flexibility improves the network structure, making it suitable for information extraction. Figure 4 shows how modularity and partitioning increase the network’s adaptability and customizability. GELAN enables several computing blocks, solving current architecture restrictions and providing a scalable deep-learning solution.

In this study, leveraging GELAN’s flexibility, we can efficiently and swiftly obtain the toddler’s body information, laying a solid foundation for subsequent head information extraction.

### 2.2. Focusing on Toddler’s Head Information

**Figure 5:**
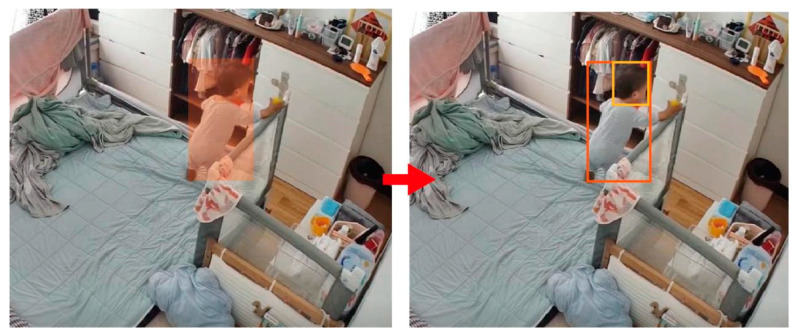
Focus on the head information.

We extract specific features from the toddler’s head after obtaining human body information using the improved YOLOv8 model, as shown in Figure 5. Head features are essential for making the final assessment.

Based on toddler body information extracted in the previous phase, we created a specialized model for recognizing heads. The model is employed for the following reasons:

1. The method is robust to image noise or pixel module processing.
2. The method boasts robust feature selection and verification capabilities, effectively preventing the extraction of non-target features. Additionally, it significantly reduces the computation time.
3. The approach is trained using the minimal entropy concept and may be readily parallelized. However, false alarms may still occur. As a result, the precise head location requires further localization.

This head-recognized model separates human body information into the head and the central torso section [29]. The fundamental concept of this algorithm involves partitioning the human body into two interconnected cubes. Both cubes have the same axis orientation along the line segment, linking the center of the head and the center of the body. This categorization reduces the intricacy of the structure of the human body but also enables the algorithm to analyze visual input that contains sequential patterns more effectively.

Given that we deal with sequential visual data, the algorithm uses spatiotemporal contextual information to reduce the search area for body detection (particularly the head). This implies that when the algorithm examines a new video frame, it anticipates where the human body will most likely be in the current frame based on information from the previous frame, limiting the search region and increasing detection efficiency.

If the initial frame is being analyzed or no toddler body is detected within the first two seconds, the search area will include the entire frame. This is due to the algorithm’s insufficient historical data to establish a search reference in such circumstances. Once the algorithm accurately identifies the location of the torso and head of the human body in the frame, it will provide the current center point position information as ${\mathrm{point}}_{i}$. This information is crucial for tracking and recognizing the body in subsequent tasks.

We recognize the algorithm’s potential for misdetection or omission by categorizing the picture characteristics using a Random Forest (RF) classifier [30]. This classification allows us to determine whether a detected feature corresponds to the head.

Furthermore, the algorithm can deal with situations where the human body cannot be precisely identified. During this procedure, the algorithm gradually increases the region of head detection. The algorithm’s capacity to dynamically update the search zone allows it to adapt to changing conditions and fluctuations in human motion successfully.

Algorithm 1 is for body and head information extraction.
**Algorithm 1.** Body Information Extraction **Inputs:** Images to be processed
 **Output:** Center of mass point K of the child’s body, center of mass point H of the head
 1. Segment the information of the toddler’s surveillance frame; 
 2. Capture the toddler’s body information and extract the centroid K; 
 3. Decompose the body information into two interconnected cubes: the head and the main torso part; 
 4. Based on the information in the in-1 frame or the in-frame, predict the possible locations of the human body in the current frame and narrow down the search range;
  **IF**:
  Human body has been detected, output center of mass point H of the head;
  **Otherwise:**

  Expanding the search area to the entire frame to look for body information that output center of mass point H of the head.

### 2.3. “Safe Zone” Demarcation

It is critical to recognize the current state of young children to provide appropriate care. However, status assessments require more than retrieving information about a child’s body and head. This is because a child’s status is influenced not just by their physical and mental health, but also by their surroundings. As a result, we suggest a solution to this problem by establishing “safe zones”.

In this study, we initially restricted the “safe zone” to the bed and sofa at home. Most toddlers in these two regions climb over guardrails, tumbling in the dataset we collected. We can improve child safety by properly labelling mattresses, couches, and guardrails.

In future studies, we should examine dining chairs, tables, and strollers to ensure toddler safety. Labeling and tracking these household objects will assist us in understanding a child’s health.

We partition the “safe region” using Hough Transform to ensure precision [31]. This method is resilient to interference and insensitive to residual straight lines, noise, and other non-linear features in the image. It is tolerant of feature border description gaps and less impacted by image noise. It may be modified to meet specific needs and applies to beds, sofas, and other home furnishings. The Hough Transform method, known for its universality and simplicity of implementation, has been widely utilized in object identification, such as hand gestures [32] and airplanes [33].

**Figure 6:**
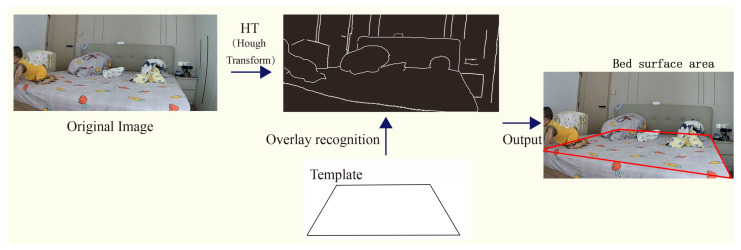
GHT-based bed surface identification.

The Hough Transform converts a curve or straight line of the same shape in one coordinate system to a point in another, creating a statistical peak problem rather than a problem of identifying arbitrary forms. The bedroom environment photos contain blankets and pillows, making it hard to locate the bed surface. We apply a Generalized Hough Transform (GHT) [34]. It can identify forms and extract the shape of the bed surface using a predefined template. Figure 6 displays the stage of GHT-based bed surface identification. It filters by the detected contour’s area to find contours representing bed surface components while avoiding contours depicting other items. The specific Algorithm 2 is displayed below.
**Algorithm 2.** GHT based bed surface detection **Inputs:** Colorful image to be detected
 **Output:** Detected bed boundary line 
  1. Convert the color image to be detected into gray scale; 
  2. Remove the high frequency signal and smooth the image by Gaussian kernel denoising; 
  3. Use gradient operator, Laplace operator, canny, and sobel for edge extraction; 
  4. Perform the edge point judgment by the principle of binarization, i.e., gray scale value = 0/255;
  5. Prepare two containers, one for displaying the Hough-space profile, an array Hough-space used to store the values of voting;
  6. Take the local maxima, set the threshold, filter the interfering straight line, draw the straight line, and calibrate the corner points.
  Output the bed boundary

### 2.4. State Label Delineation

**Figure 7:**
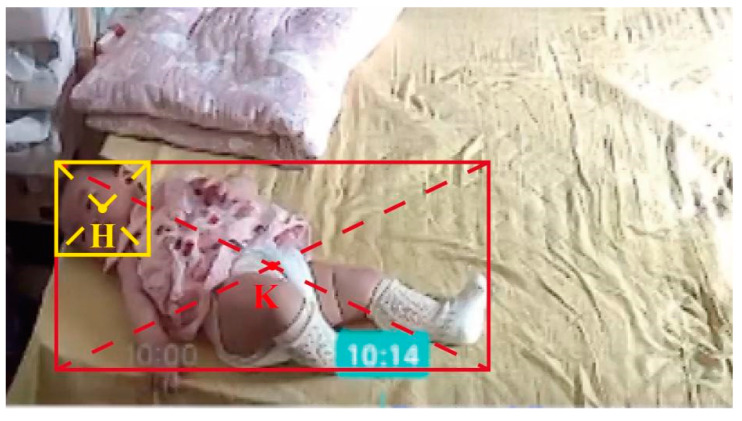
Head and body center of gravity points.

Based on the previously extracted body feature information, we obtain the center of mass point K of the toddler’s body and the center of mass point H of the head, as shown in Figure 7.

We added the safety icon $S_{p}$. We identify the toddler as $S_{1}$ while they are safe, when they may fall, and $S_{3}$ when they have already fallen. Security symbol division is shown clearly in Algorithm 3.
**Algorithm 3.** Security Symbol Division **Inputs:** center of mass point K of the child’s body, center of mass point H of the head, safety zone $S_{p}$
 **Output:** safety markers *S*_1_, *S*_2_, *S*_3_
 **If:**
 H is within $S_{p}$, then it is currently in *S*_1_;
 Point H leaves the region, and K is within $S_{p}$, the current output is *S*_2_;
 **Otherwise:**
 The output is *S*_3_.

According to the algorithm above, we established a particular determination technique for the division of the child’s state and the safety mark, representing the three states in which the kid is. The precise delineation of the circumstances and the basis are shown in Table 1.

## 3. Experiments and Results

We performed experiments on our designated datasets to fully illustrate the efficacy of our suggested detector and approach. The experimental results validate the efficacy of our approach.

### 3.1. Dataset

**Figure 8:**
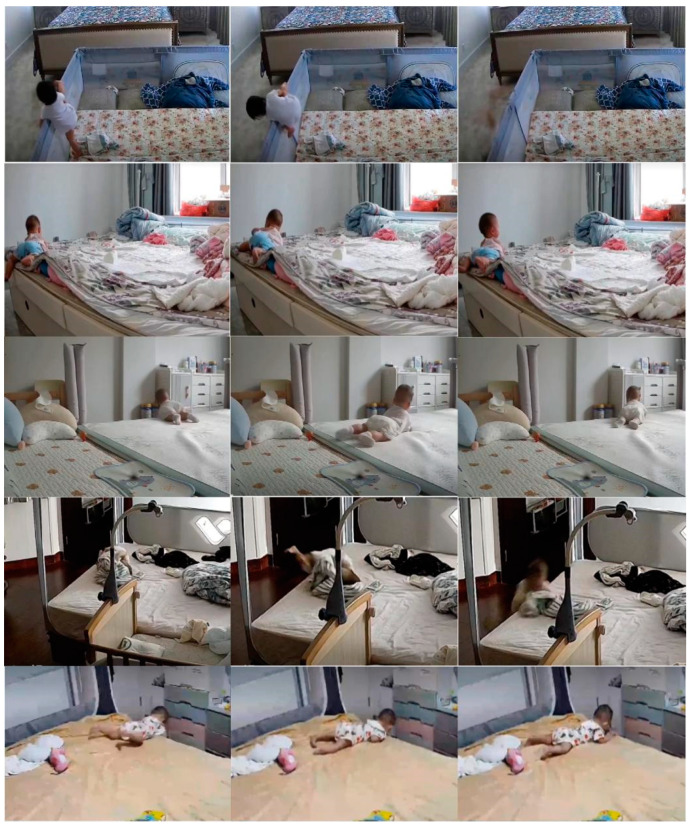
Images in self-built fall dataset of toddlers.

To assess the validity of the proposed methodology, we used fall events as test cases. We collected 230 videos of toddlers falling in the home environment from parents. These videos focused on the daily activities of toddlers between 13 and 30 months of age, particularly when they fell from their beds or sofas. We included 108 toddlers in our sample pool and covered diverse lighting conditions, camera settings, and indoor furniture layouts. These measures were intended to enhance the breadth and representativeness of the dataset, and some of the representative data are shown in Figure 8.

Each video lasted 10 s to 10 min and vividly captured the toddler’s daily activities, including falls on the bed or sofa. These show toddlers’ daily activities, such as falling forward or backwards on the bed or being held by parents after falling without hitting the ground. Based on this rapid fall movement of the head, we carefully selected two to three clips for each video.

Subsequently, we take 500 video clips (×30 FPS) extracted from the 200 videos into a training set and a validation set in the ratio of 8:2. In addition, a separate test set of 100 video clips (×30 FPS) cut from another 30 videos concentrating on children’s falling behaviors at home without direct adult supervision was created to completely evaluate our framework’s effectiveness.

The experimental model operates on Windows 11 Professional Edition (Microsoft Corporation, Redmond, WA, USA). It was configured with a CPU: Intel i5 13600KF (Intel Corporation, Santa Clara, CA, USA) and GPUs: Nvidia GTX 4070 Ti 12 GB (Nvidia Corporation, Santa Clara, CA, USA), employing Python 3.8, PyTorch 2.0.1, and cuDNN 8.9.1.

### 3.2. Experimental Setting

Considering the dataset size and training efficiency, we set the batch size to 16 and planned 300 Epochs for training. All photos within the dataset were normalized to 640 × 640 pixels and transmitted over the network to train the extraction model. The initial learning rate was set to 0.01, while the final learning rate was adjusted to 0.10 to optimize the training process. For the toddler information model, we specifically used images scaled to 300 × 300 pixels from the dataset for training. In addition, a weight decay coefficient of 0.0005 was carefully set, and the learning rate was defined to vary from 0.0002 to 0.02, aiming to refine the model’s training effect further. To comprehensively and accurately evaluate the performance of our model on the task of classifying the state of a toddler, we set up four clear and mutually exclusive result classifications, which cover all possible scenarios as follows:

The first is that the system correctly identifies the toddler’s current state.
The second is that an event that did not result in a fall was wrongly classified as an alarm state.
The third one is that no fall occurs; hence, the algorithm does not segment it.
The fourth is a fall event that the system does not recognize.

Based on the four different scenarios mentioned above, we segmented them into four categories: TP, FP, TN, and FN, using a specialized measurement method.

True Positive (TP): A fall occurred, and the system accurately classified the fall.
False Positive (FP): The fall did not occur, but the system misclassified it.
True negative (TN): No fall occurred, and the system accurately identified it.
False negative (FN): A fall occurred, but the system incorrectly labeled it.

The evaluation of classification models is fundamental to machine learning. Model selection and performance evaluation frequently use numerous indicators for a holistic approach. These numbers show the model’s classification accuracy and performance in different settings. In practice, we must choose evaluation metrics based on task needs and background information.

### 3.3. GELAN Improved Results

To verify the enhanced performance of YOLOv8 on our custom dataset, we conducted a comparative analysis with the original YOLOv8 model, YOLOv3, YOLOv5, and YOLOv6. The results in Table 2 show that YOLOv3 and YOLOv5 have poor detection performance in the toddler head and body information extraction scenario, barely reaching about 85%. In terms of metrics, the improved YOLOv8 network uses GELAN and has a mAP@50 score of 97.26%; the average accuracy is 11.65, 17.32, 7.26, and 3.15 percentage points higher than that of YOLOv3, YOLOv5, YOLOv6, and YOLOv8, respectively. All data in the table are mean values. Regarding accuracy and recall, the improved YOLOv8 model achieves 96.33% and 97.87%, respectively, higher than the original YOLOv8 model. In addition, the FPS of the enhanced algorithm is 69.97, and the improved model is more suitable for small target detection.

The improved YOLOv8 model proposed in this paper achieves significant performance gains in head and body information extraction from toddlers by replacing the original c2f module with the GELAN module. This performance improvement is mainly due to the following reasons:

1. More efficient feature extraction: The GELAN module combines the advantages of multiple network structures to enhance the model’s ability to recognize toddlers’ body features through effective hierarchical aggregation and the combination of CSPNet and ELAN. This combination not only enhances the richness of the features, but also speeds up the feature extraction by keeping its time difference within 3 s.
2. Adaptable: The improved YOLOv8 model is optimized for complex background and small target detection. The flexibility of the GELAN module allows the network to better adapt to the characteristics of young children’s large changes in body size and dynamics, which improves the robustness of the detection and increases the accuracy by nearly 20% compared to the original YOLO model.
3. Optimized network structure: GELAN reduces the computational burden of the model through more efficient data processing and simplification of the network structure, while maintaining high accuracy with an output frame rate of 69.97 FPS, which makes the model more suitable for running on resource-limited devices and improves the model’s practicality and scalability.

### 3.4. Human Information Extraction Results

To evaluate the effectiveness of each daily activity sample in the self-built dataset, we randomly selected 15 discontinuous frames from each video clip. There are a total of 7500 frames (15 × 500) for performance evaluation. We identified and labeled the toddler body information for each frame in boxes. The selected frames will be divided into an 8:2 ratio for training and testing. Among them, the training set randomly selected proportionally is used for Random Forest (RF) training and the rest are tested. The following criteria are used to evaluate the accuracy of human body and head information extraction:

(1) $$T_{i}=\left\{\begin{array}{c} r_{i}<\lambda ,\;0\\ r_{i}\geq \lambda ,\;1 \end{array}\right.$$

where Ti denotes whether the toddler body and head information has been successfully extracted in the frame; ri denotes the intersection of the extracted human body and head information rectangles, and the actual information rectangles; and λ is a predefined threshold. Our rule states that the higher λ is, the more stringent the criteria for effectively extracting the toddler`s upper body. If ri is less than λ, it indicates that the toddler body information extracted in the frame is inaccurate and outputs it as 0. On the contrary, it suggests that it is accurate and outputs it as 1. Given that it is impossible to achieve a 100% accurate rate in the actual application of the algorithm, we can disregard the effect of the error on the results when the intersection between the measured and actual information reaches a specific value. We first converted the image to be measured into a histogram, essentially the probability density distribution of points with varying pixel values. Each bar in the histogram is divided by the total number of points to obtain the a priori probability of its occurrence, which equals one. Then, the image is traversed, separating the pixel points into two portions, A and B, and calculating the sum of the probability of occurrence of A and B pixels, the mean UA, UB, and their respective variances, sigmaA and sigmaB. All acquired parameters are combined to form an objective function, and the minimum objective function is used to determine the suitable threshold. The calculation above yielded the algorithm’s threshold value λ to be 0.8.

(2) $$A=\frac{\sum_{i=1}^{n}r_{i}}{n}$$

**Figure 9:**
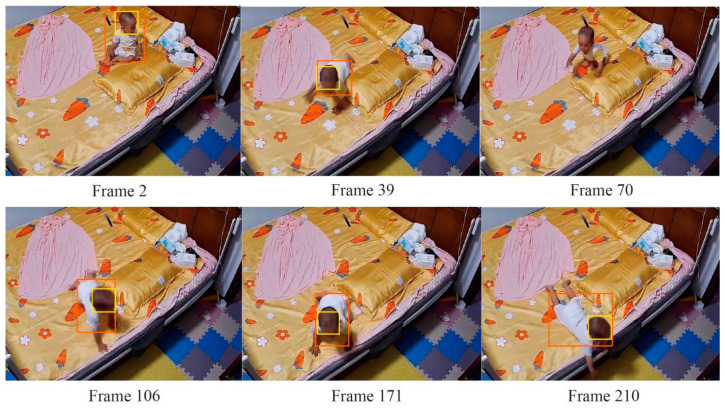
Live examples of toddler body extraction.

In the above Equation (2), A is the accuracy of the whole extraction result and n is the total test sample size. The final extracted result is 95.3%, and we can conclude that the method has a strong advantage for extracting human body and head information. At the same time, the method has a strong learning ability. If the human body information in a certain frame is missing, it can still extract the human body information in the next frame efficiently through learning. Figure 9 shows the results of the method in the inspection of the image, as shown in the figure in the 70th frame, the detection of the omission. However, the subsequent detection is not affected by the still accurate extraction.

To highlight the superiority of our toddler upper body identification approach, we compare it with the popular methods. Zhao et al. [35] used effective human upper body detection method to extract the center of the human head and upper body by using a RF approach. Shen et al. [12] extracted LTDP visual descriptors for human body detection and combined with SVM classifier. The final performance comparison results are listed in Table 3. We can clearly observe that the human head and body information extraction method by improving YOLO is greatly superior to the methods of Zhao and Shen. We can observe that these two comparison methods cannot handle the human–bed position interactions and substantial postural fluctuations that occur during the fall-from-bed phase, resulting in unsatisfactory human body extraction results.

### 3.5. State Classification Results

We tested the model’s reliability using 300 additional video clips of toddlers falling at home. We renamed the safe, warning, and hazardous as states 1, 2, and 3, respectively, resulting in two out of sixty safe state samples being misdiagnosed as warning states and one out of sixty hazardous state samples. In contrast, three of the ninety-six warning state samples and five of the one hundred forty-four danger state samples were misdiagnosed as safe. Table 4 displays the number of young children identified in various states.

**Figure 10:**
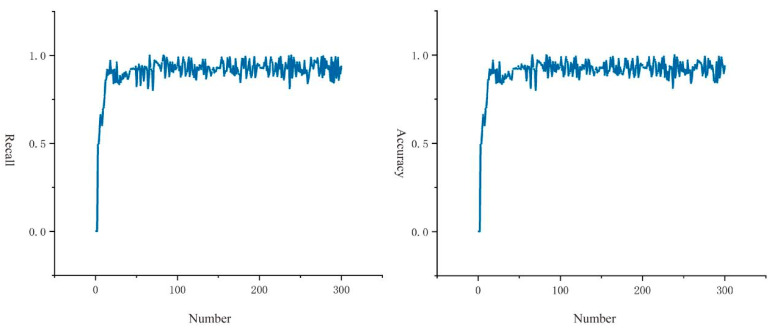
Recall and accuracy curves for 300 test samples.

The accuracy and recall curves of the alarm algorithm studied in this paper are shown in Figure 10.

We can see that both values remain over 0.84 during the training process, and practically all of them are close to one. Based on YOLO enhancement, it demonstrates that our suggested fall alarm algorithm performs well and can play an important role in state recognition and alert, with the specific formula presented in Equation (3).

(3) Re⁡call=TPTP+FN

This study evaluates the algorithm’s overall performance, which is displayed in Table 5. The precision of state classification for toddlers is 98.72%, with an accuracy rate of 96.33%. This implies that the method presented in this study may efficiently identify the current condition of the toddler body with a low leakage detection rate. Meanwhile, its specificity is 95%, and its f-score is 97.68%, indicating that the approach can distinguish between falling and daily activity behavior with fewer false positives.

## 4. Discussion

Based on its structure and performance, the detection software proposed in this work has the potential to be extended to public settings, as proved by the task of detecting toddlers falling. The detection technique is separated into two steps, aiming to accurately and conveniently detect toddler falling in real-time. In stage I, the features of the toddler’s body and head are separately extracted. In stage II, position status detection is conducted on the retrieved data using the “safe zone”. The experiment results suggest that the framework can reduce false and missed detections in complex household settings compared to other initial YOLO models.

The suggested detection system can be expanded functionally and architecturally to encompass outdoor situations. For instance, at community playgrounds, surveillance cameras near slides and climbing frames can effectively monitor cases of falls and promptly send real-time alert warnings. It can also be extended to help with recreational facilities for the elderly [36].

While our technique yields good detection outcomes, it also exhibits limitations.

1. The computer’s performance influences the computing time of the recognition algorithm in this article. In practice, computing platforms may behave differently from our specific setting in research. The algorithm’s GPU usage, memory requirements, and energy consumption will be assessed in our future work to discover resource bottlenecks. Based on the evaluation results, we will incorporate lightweight optimization strategies like model trimming and quantization to make the algorithm perform efficiently on traditional CPUs and edge devices utilized in real-time applications. These changes will improve our research’s practicality and sustainability.
2. We did not examine the safety zone of other pieces of furniture, such as dining chairs, dining tables, strollers, and other surfaces that toddlers frequently interact with and are at a high risk of falling from. Our future research should focus on expanding this further.
3. Based on the error results of our detection, we should develop corresponding correction mechanisms. Firstly, certain toys and patterns may still be mistaken for toddlers, leading to error detection. These confused objects are so close to toddlers in texture, color, and form that even video viewers find it difficult to distinguish between them. To effectively filter out small noises, the algorithm requires more optimization. Secondly, our algorithm is sensitive to noise caused by unusual photos and changing scene conditions. When confronted with a situation in which a toddler is in bed with a quilt covering the body, the system fails to capture the physical features. Thirdly, The RGB camera-based method remains unrecognizable due to excessively dim illumination. We should enhance the technique’s capacity to identify tiny, illegible targets, adjust model parameters, and enhance the complexity of model training.

## 5. Conclusions

This study suggests a toddler fall alarm approach based on the improved YOLOv8 model. The detection technique is divided into two steps. The first stage begins with extracting video frames, and the toddler body feature information is gathered by processing the surveillance video captured by improved YOLOv8. The toddler’s body feature data from the previous phase is then processed and incorporated using a head capture technique. In the second stage, the “safe zone” is computed using GHT, and the spatial location is compared to the two centers of mass points, K for the toddler’s body and H for the head from the previous stage. Position status detection is performed on the extracted data. The experimental findings demonstrate that the proposed framework outperforms YOLOv3, v5, v6, and v8 regarding detection precision, recall, mean average precision, and F1 scores. Additionally, it meets the typical FPS requirement for general surveillance cameras. It has an accuracy of 96.33 percent, a sensitivity of 96.67 percent, and a specificity of 95 percent.

## Figures and Tables

**Table 1:** Child status division.

Position Relation	Safety Symbol	State Type
H ∈ *S_p_*	*S*_1_	security status
H ⊄ *S_p_*, K ∈ *S_p_*	*S*_2_	alarm status
H ⊄ *S_p_*, K ∈ *S_p_*	*S*_3_	dangerous status

**Table 2:** Comparison of detection performance of different YOLO models.

Model	P	R	F1	mAP@0.5
YOLOv3	0.8394	0.8222	0.7903	0.8561
YOLOv5	0.7699	0.7810	0.7801	0.7994
YOLOv6	0.8588	0.8817	0.8591	0.9
YOLOv8	0.905	0.9271	0.9211	0.9411
Improved YOLOv8	0.9491	0.9553	0.9572	0.9726

**Table 3:** Human body extraction results.

**Research**	Zhao et al. [35]	Shen et al. [12]	Ours
**Accuracy**	0.78	0.73	0.953

**Table 4:** Child status identification results.

Safety Status	Warning Status	Dangerous Status
60	96	144

**Table 5:** Results of the infant status classification.

Sensitivity	Specificity	Accuracy	Precision	F-Value
96.67%	95%	96.33%	98.72%	97.68%

## Data Availability

Guardians of the toddlers in the collected dataset have requested that the dataset not be made publicly available.

## Abbreviations

The following abbreviations are used in this manuscript:

SDI	Shuffle Dimensionally Integrated
ELAN	Long Short-Term Memory Efficient Layer Aggregation Networks
SPPCSPC	Spatial Pyramid Pooling, Cross Stage Partial
STGCN	Spatio-Temporal Graph Convolutional Network
ECA	Efficient Channel Attention
SSD	Single Shot Detector
ADLs	Activities of Daily Living
GELAN	Generalized Efficient Layer Aggregation Network
CSPNet	Cross Stage Partial Network
GHT	Generalized Hough Transform
RF	Random Forest
TP	True Positive
FP	False Positive
TN	True Negative
FN	False Negative
